# Testing the Pauli Exclusion Principle across the Periodic Table with the VIP-3 Experiment

**DOI:** 10.3390/e26090752

**Published:** 2024-09-02

**Authors:** Simone Manti, Massimiliano Bazzi, Nicola Bortolotti, Cesidio Capoccia, Michael Cargnelli, Alberto Clozza, Luca De Paolis, Carlo Fiorini, Carlo Guaraldo, Mihail Iliescu, Matthias Laubenstein, Johann Marton, Fabrizio Napolitano, Kristian Piscicchia, Alessio Porcelli, Alessandro Scordo, Francesco Sgaramella, Diana Laura Sirghi, Florin Sirghi, Oton Vazquez Doce, Johann Zmeskal, Catalina Curceanu

**Affiliations:** 1 Laboratori Nazionali di Frascati, Istituto Nazionale di Fisica Nucleare (INFN), Via E. Fermi 54, I-00044 Rome, Italy; massimiliano.bazzi@lnf.infn.it (M.B.); nicola.bortolotti@cref.it (N.B.); cesidio.capoccia@lnf.infn.it (C.C.); alberto.clozza@lnf.infn.it (A.C.); luca.depaolis@lnf.infn.it (L.D.P.); guaraldo@lnf.infn.it (C.G.); mihai.iliescu@lnf.infn.it (M.I.); johann.marton@oeaw.ac.at (J.M.); fabrizio.napolitano@lnf.infn.it (F.N.); kristian.piscicchia@cref.it (K.P.); alessio.porcelli@oeaw.ac.at (A.P.); scordo@lnf.infn.it (A.S.); francesco.sgaramella@lnf.infn.it (F.S.); sirghi@lnf.infn.it (D.L.S.); fsirghi@lnf.infn.it (F.S.); oton.vazquezdoce@lnf.infn.it (O.V.D.); catalina.curceanu@lnf.infn.it (C.C.); 2 Centro Ricerche Enrico Fermi, Museo Storico della Fisica e Centro Studi e Ricerche “Enrico Fermi”, Via Panisperna 89a, I-00184 Rome, Italy; 3 Stefan-Meyer-Institute for Subatomic Physics, Austrian Academy of Science, Kegelgasse 27, 1030 Vienna, Austria; michael.cargnelli@oeaw.ac.at (M.C.); johann.zmeskal@oeaw.ac.at (J.Z.); 4 Dipartimento di Elettronica, Informazione e Bioingegneria and INFN Sezione di Milano, Politecnico di Milano, I-20133 Milano, Italy; carlo.fiorini@polimi.it; 5 Laboratori Nazionali del Gran Sasso, Istituto Nazionale di Fisica Nucleare (INFN), Via G. Acitelli 22, I-67100 L’Aquila, Italy; matthias.laubenstein@lngs.infn.it; 6 Institutul National Pentru Fizica si Inginerie Nucleara Horia Hulubei (IFIN-HH), Str. Atomistilor No. 407, P.O. Box MG-6, RO-077125 Magurele, Romania

**Keywords:** PEP, VIP-3, MCDF

## Abstract

The Pauli exclusion principle (PEP), a cornerstone of quantum mechanics and whole science, states that in a system, two fermions can not simultaneously occupy the same quantum state. Several experimental tests have been performed to place increasingly stringent bounds on the validity of PEP. Among these, the series of VIP experiments, performed at the Gran Sasso Underground National Laboratory of INFN, is searching for PEP-violating atomic X-ray transitions in copper. In this paper, the upgraded VIP-3 setup is described, designed to extend these investigations to higher-Z elements such as zirconium, silver, palladium, and tin. We detail the enhanced design of this setup, including the implementation of cutting-edge, 1 mm thick, silicon drift detectors, which significantly improve the measurement sensitivity at higher energies. Additionally, we present calculations of expected PEP-violating energy shifts in the characteristic lines of these elements, performed using the multi-configurational Dirac–Fock method from first principles. The VIP-3 realization will contribute to ongoing research into PEP violation for different elements, offering new insights and directions for future studies.

## 1. Introduction

The Pauli exclusion principle (PEP) stands as a foundational pillar of quantum mechanics, stating that in a system, two identical fermions cannot share identical quantum numbers [1,2]. The PEP’s consequences have profound effects in several sectors, from the singlet nature of helium’s ground state to the stability of matter [3] or the microscopic description of superconductivity and neutron stars [4].

The PEP is theoretically grounded in quantum field theory, as directly proven by Pauli [5]. The subsequent developments led to the spin-statistics theorem, which imposes constraints on the particle exchange symmetry of the total wavefunction based on the particle’s spin [6]. For particles with half-integer spin, the theorem mandates the antisymmetry of the total wavefunction, defining the properties of fermions. Conversely, the symmetric case defines bosons with integer spin. In the context of quantum field theory, this symmetry defines the commutation rules of field operators by determining their anticommutator (commutator) for fermions (bosons). Written in these mathematical terms, the connection is clear with the early postulate of asserting the impossibility to have fermions with the same all quantum numbers. Attempts to formulate a theory that generalizes these commutation rules have been investigated over the years. This finds application, for example, in the case of quasiparticles with anyons [7], which possess a general quantum commutation with a phase and are observed in a two-dimensional semiconductor [8].

Ignatiev and Kuzmin [9] provided a model that includes a nonzero probability amplitude for two identical fermions to occupy the same state. This model realizes the PEP violation by modifying the anticommutation relations for the creation and annihilation of fermions and delineating the algebra necessary to support such a change. Subsequent efforts by Greenberg and Mohapatra [10,11] employed the quon model to incorporate violations of the Pauli exclusion principle (PEP) within a quantum field theory framework. Since these developments, the PEP violation probability is denoted as $\beta^{2}/2$.

Messiah and Greenberg (MG) showed [12] that even within a theoretical framework that allows for a violation of the spin-statistics connection, in a given system, transitions between states with different symmetries are forbidden. This superselection rule proposes to perform experimental verifications of the spin-statistics connection by introducing new fermions in a pre-existing system of fermions and test the newly formed symmetry state. The pioneering experimental approach adopted by Ramberg and Snow [13] was to circulate a direct current in a copper target and to search for PEP violating atomic transitions of these electrons. In case of a violating transition, a shift should be observed in the emission from the 2p to the 1s level of the metal. This shift reduces the normal transition energy because of the additional electronic screening present in the final state. The shift can be ab initio calculated with the multi-configurational Dirac–Fock (MCDF) method [14]. Indeed, the transition energy that violates symmetry is equal to the energy difference between the initial and final states when the violating electron is not antisymmetrized with respect to the total wavefunction. In the case of copper, such an energy shift is about 300 eV [15] lower than the normal Kα line.

Following the approach by Ramberg and Snow, the VIP collaboration has improved the value of $\beta^{2}/2$ by limits on 400 [16]. The VIP series of experiments was performed at the Gran Sasso Underground National Laboratory of INFN, and, due to the low-background environment, aimed to gradually improve constraints on the value of $\beta^{2}/2$.

In this work, we introduce the developments towards the VIP-3 experiment and its advancements with respect to VIP-2 in order to study PEP violations for different elements in the periodic table. The VIP-3 experimental apparatus is showcased with all the improvements towards measuring heavier elements compared to copper in VIP-2. The experimental effort is supported by ab initio calculations of PEP-violating shifts for the X-ray lines of interest with state-of-the-art first-principle code.

The paper is structured as follows: in Section 2, we briefly review the VIP-2 experimental setup and present the VIP-3 experimental design setup with the relative improvements. In addition, details of the performed MCDF calculations are presented. In Section 3, we present the VIP-3 components’ characterization towards the detection of PEP-violating lines across the periodic table, together with the calculated shift for the target elements used by VIP-3. Section 4 concludes the paper.

## 2. Setups and Methods

The VIP experiments are performed at the Gran Sasso Underground National Laboratory (LNGS) of INFN. The underground laboratories’ low background enables us to perform experiments searching for very weak signals. Indeed, the underground location, equivalent to being under 3600 m of water, reduces the cosmic ray flux rate by about six orders of magnitude, significantly enhancing the experimental sensitivity conditions.

**Figure 1:**
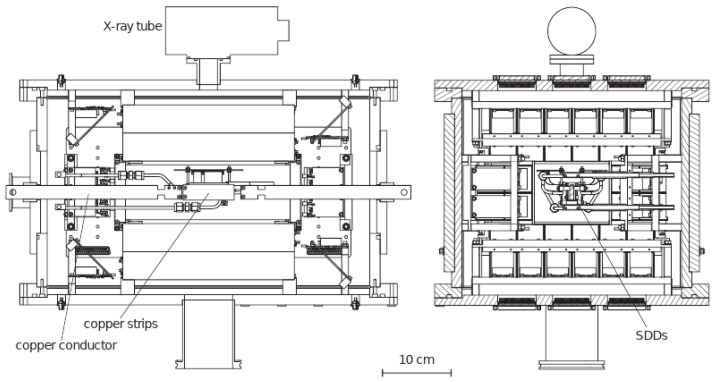
VIP-2 Setup. The lateral (**left**) and front (**right**) sections of the VIP-2 apparatus. In the lateral section, the copper targets, where the current circulates, are indicated alongside the X-ray tube used for internal calibration. In the front section, the SDD detectors are highlighted within the vacuum chamber. Image reproduced from [18].

In contrast to the VIP experiment that utilized charge coupled devices (CCDs) for X-ray detection [16], VIP-2 (see Figure 1) employs silicon drift detectors (SDDs) developed in collaboration with Politecnico di Milano (PoliMi, Milan, Italy) and the Fondazione Bruno Kessler (FBK, Trento, Italy). These SDDs feature a thickness of 450 μm with a large geometrical acceptance due to the overall active area of 0.64 cm^2^. In terms of spectral response, they have an energy resolution of 190 eV (FWHM) [17], and an efficiency of 99% at 8 keV, which is the energy of interest of the VIP-2 experiment.

**Figure 2:**
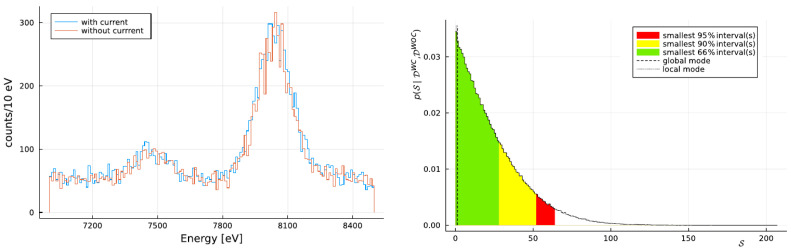
VIP-2 results. Main results of VIP-2, with the spectra shown on the (**left**) without (orange) and with (blue) current, and the marginalization of the violation amplitude, as determined from the Bayesian analysis in [18], displayed on the (**right**).

During the data taking, periods with current on (signal) and current off (background) are alternated. A current of 180 A circulates through a pair of copper targets, each with dimensions of 71 mm in length, 20 mm in height, and 25 μm in thickness. Due to the high current circulating inside the targets, they are kept at a constant temperature of 15 °C with a dedicated water cooling system. The SDDs are operating at −90 °C and positioned adjacent to the pair of targets. In the period from 2016 to 2017, there were two arrays of 1 × 3 SDDs, whereas starting from 2018, there are four arrays of 2 × 4 SDDs. Within the vacuum chamber, an Fe-55 source, encapsulated by a titanium foil, is utilized for internal calibration. The operational pressure of the vacuum chamber is maintained at 10^−5^ mbar. The spectra obtained with current on and off and marginalization of the amplitude from the VIP-2 analysis [18] are shown in Figure 2.

**Figure 3:**
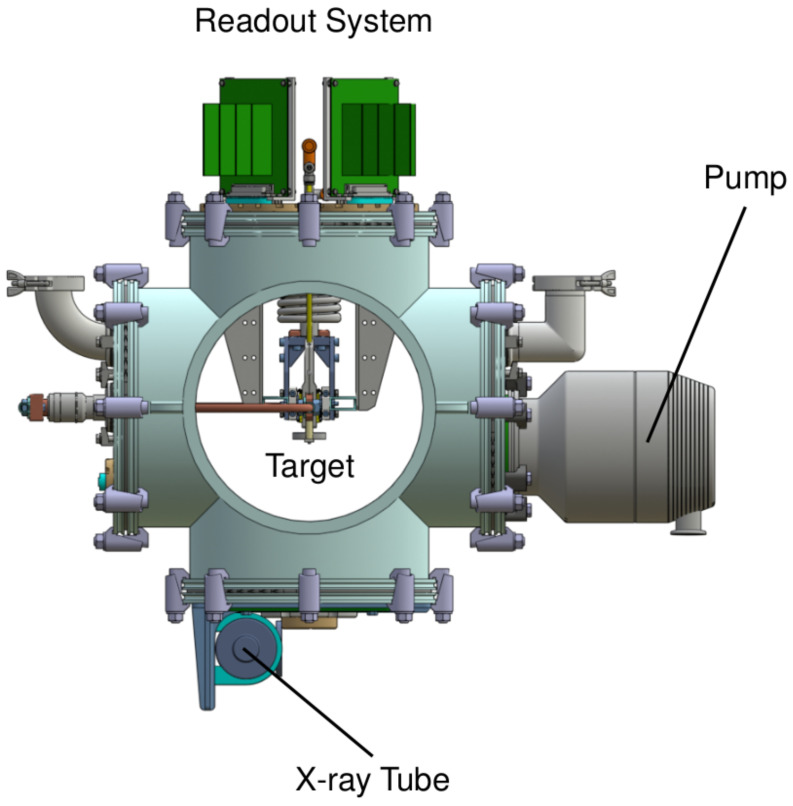
VIP-3 Setup. The VIP-3 vacuum chamber, where the pump, X-ray tube, readout electronics, and target are highlighted. The front and rear flanges are removed to visualize the target more clearly.

VIP-3 is aiming to extend the investigation of Pauli-violating transitions to different targets made of heavier elements compared to copper. The design of the new setup is outlined in Figure 3, where the core elements of the experiment are highlighted.

The newly designed vacuum chamber is connected to the pump for operation in high vacuum (10^−5^ mbar). The calibration is performed similarly to VIP-2, utilizing an internal Fe-55 source in conjunction with an X-ray tube positioned beneath the vacuum chamber, exciting metallic foils for calibration. The readout electronics are mounted on top, and the front-end electronics offer improved performance in terms of linearity, stability, and gain.

Inside the VIP-3 vacuum chamber, the target is placed alongside the SDDs, and attached to this is the cooling system to maintain a working temperature for the SDDs of −140 °C. The thermal contact between the cold fingers and the SDD cooling block will be made of pure copper, to reduce natural copper radio contamination. Utilizing thermal contacts and the cooling block in copper offers the advantage of exploiting the higher thermal conductivity of copper, as opposed to the steel used in VIP-2. This aspect will result in reducing the working temperature, enhancing the energy and timing resolutions, and potentially increasing the circulating current intensity up to 400 A. The targets will be made of silver, tin, zirconium, and palladium.

**Figure 4:**
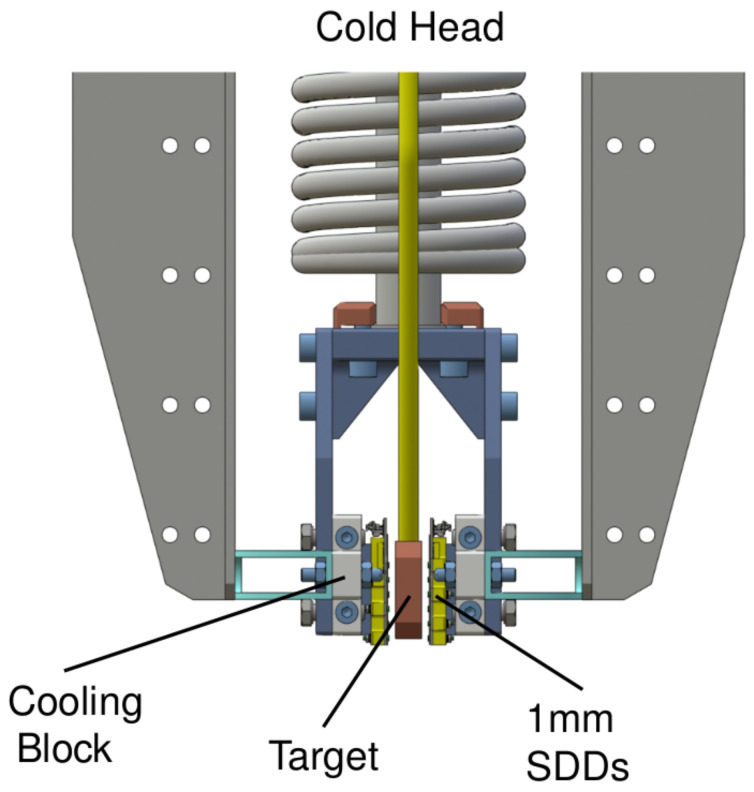
Inner configuration of VIP-3. Cross-sectional illustration of the VIP-3 experimental apparatus, showcasing the target material, surrounded by the thicker SDDs for enhanced data collection and the comprehensive cooling block system for optimized temperature regulation.

**Figure 5:**
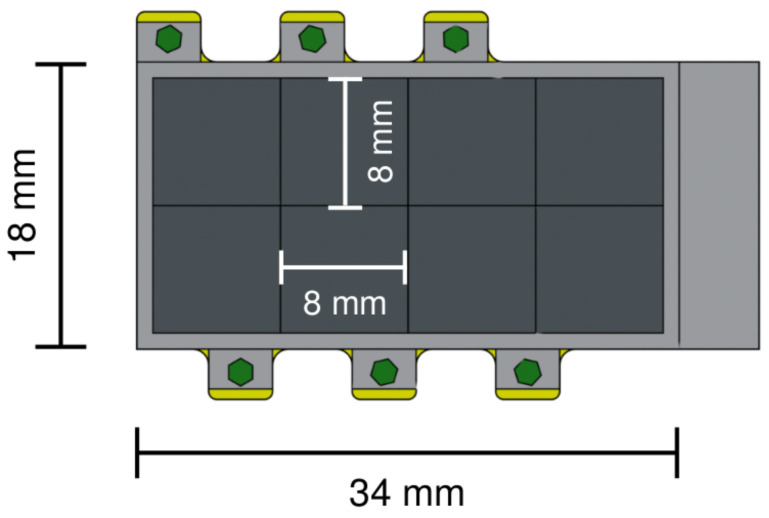
Design of the SDD array. Top view of the thicker SDD array, detailing the overall dimensions of the unit and with individual measurements for each pixel unit within the array.

The SDDs will be positioned alongside the targets to enhance the geometrical efficiency, as depicted in Figure 4, which focuses on the target region of the setup. The most significant improvement with respect to VIP-2 is the utilization of thicker SDDs measuring 1 mm, which offer increased efficiency for detecting X-ray transitions in heavier elements at higher energies. The performance of these new 1 mm SDDs is discussed in the following section. Every single SDD unit will have dimensions of 8 × 8 mm^2^, featuring a guard ring to reduce the charge-sharing effect at the edge of each unit [19], for a total area of the SDD array of 34 × 18 mm^2^. Each SDD unit consists of a 4 × 2 matrix, and VIP-3 will utilize 8 SDD arrays, enhancing the geometrical efficiency by a factor of 2 compared to VIP-2. The design of the single SDD array unit, with the overall dimension of the array, is shown in Figure 5.

Calculations of the normal and PEP-violating emission lines are performed with the multi-configuration Dirac–Fock for general matrix elements (MCDFMGE) code, version 2022.3, using the 2021 CODATA fundamental constants [20]. This ab initio code, developed starting in the early 1980s by Desclaux and Indelicato [14,21], is based on the Dirac–Fock method to find a variational approximation to the electron wave function, starting from hydrogen-like basis functions. This is performed with the constraint that the wave function is antisymmetrized with respect to all the electrons in the system and performs the self-consistent cycle where the variational parameters are optimized. The total energies are then obtained from the total wave function, and the difference between the initial and final states gives the transition energies under analysis. The Pauli-exclusion-principle-violating electron (PVE) is included by treating it in a different way. Indeed, the total wave function is still antisymmetrized with respect to the normal electrons, but it is not antisymmetrized with respect to the PVE. This is performed by including a test particle in the code with spin one-half, usually implemented for calculations with muons but, in this case, with the same mass as the electron.

As the PVE is seeing a doubly occupied final state, the transition energy is reduced due to the screening effect of the extra electron, with transition energies similar to those of a Z-1 atom for an atom with Z [15].

## 3. Results and Discussion

Searching for PEP-violating transitions across various elements is crucial for obtaining a more comprehensive understanding of quantum mechanics. As Okun highlighted [22], these tests are of special interest because they rigorously challenge and validate the fundamental principles governing the behavior of particles. Furthermore, studying emission lines in elements heavier than copper possesses a technical advantage too. Indeed, as the energy of these lines increases with Z^2^ according to Moseley’s law [23], more energetic lines suffer less absorption and are more easily detectable.

**Figure 6:**
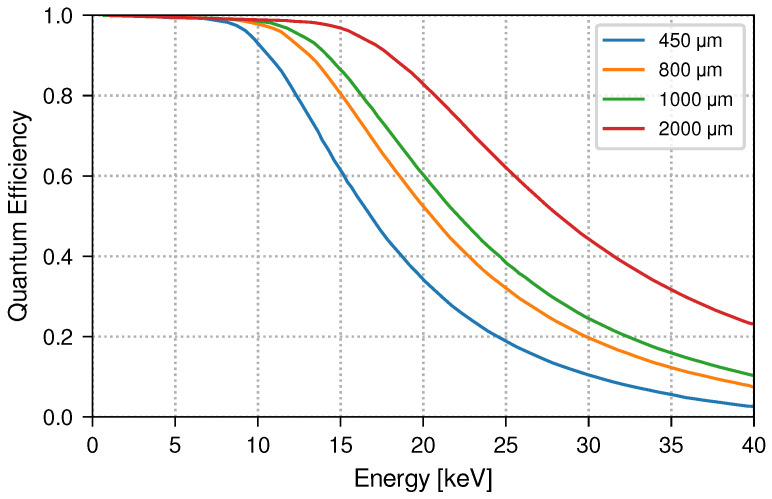
Quantum Efficiency Variation with SDD Thickness. Relationship between the quantum efficiency of SDDs and the X-ray energy for different detector thicknesses, where VIP-2 is currently using a 450 μm thickness (blue), while VIP-3 is planning 1000 μm (green).

The materials that will be used as targets are zirconium, silver, palladium, and tin. These materials have normal Kα lines at 15.7 keV, 22.1 keV, 21.1 keV, and 25.2 keV, respectively. This poses the challenge of improving the efficiency of the SDDs to effectively measure the regions around such lines. The most important factor that determines the SDD’s efficiency is the thickness of the single SDD unit. VIP-2 is currently employing SDDs with a thickness of 450 μm, and their efficiency is shown in Figure 6 in blue. It is clearly seen how the efficiency decreases when moving from the energy of interest of VIP-2 for copper at 8 keV to the case of the other elements above 20 keV. For this reason, VIP-3 is employing thicker SDDs of 1000 μm, which improves their overall efficiency at such higher energies. Additionally, as at this energy the efficiency is still lower than that for copper in the case of VIP-2, the increased operating current of VIP-3 is expected to counterbalance this effect as it will increase from 150 A to 400 A. The new thicker SSD is guaranteed to also have the same spectral feature as the previous SDDs in terms of energy resolution with 190 eV at 8 keV [17].

PEP-violating transitions in heavier elements also have the advantage that the forbidden transition is more separated from the normal Kα line. The increase in the energy shift is attributed to the fact that as the atomic number increases, the electronic screening is also enhanced due to the larger number of electrons in the atom.

We employed the MCDF method to calculate the energies of the violating transitions in the elements that will be employed by VIP-3. We first calculated the normal Kα transitions, followed by the calculations that incorporate the PVE.

The MCDF calculations for all the elements were performed by selecting the closest closed-shell configuration compatible with the observable oxidation state of the given metal, except for palladium, where the neutral state was used. This choice was made to help the convergence of the MCDF calculations, as they become more challenging to converge for open-shell systems [24]. The oxidation state and the relative electronic configuration employed are reported in Table 1, where the energy shifts for $\alpha_{1}$ and $\alpha_{2}$ lines are reported. Changing the configuration might affect the transition energy by a few eV, but the energy resolution of the SDDs is well above the eV range, i.e., 140 eV at 8 keV and not able to resolve $\alpha_{1}$ and $\alpha_{2}$ lines.

## 4. Conclusions

In this paper, we discuss the VIP experiments that aim to search for violations of the Pauli exclusion principle (PEP) across various elements in the periodic table by chasing forbidden transitions in X-ray emission spectra. VIP has progressively established stringent limits on the parameter $\beta^{2}/2$, which quantifies the probability of the violation. Here, we introduce VIP-3, which extends the methodology to heavier elements beyond the scope of VIP-2, which is focused only on copper. VIP-3 will employ zirconium, silver, palladium, and tin as target materials and features a newly developed setup designed to handle and measure these heavier elements. Significant advancements include the use of thicker silicon drift detectors, which will enhance the sensitivity to more energetic X-rays emitted from these heavier elements. Additionally, we performed calculations from first principles using the multi-configuration Dirac–Fock method to predict energy shifts for the violating transitions in these new targets.

The VIP-3 setup is under construction, to be installed and start data acquisition at LNGS-INFN in 2025, aiming to offer new insights into theories allowing PEP violation.

## Figures and Tables

**Table 1:** Energy and Shift values for PEP-Violating Transitions. Values of the transition energy and shift with respect to the normal lines for K$\alpha_{1}$ and K$\alpha_{2}$ for different elements, where the electronic configuration employed is also reported in the first column.

Element	Configuration	K$\alpha_{1}$ [eV]	K$\alpha_{1}$ Shift [eV]	K$\alpha_{2}$ [eV]	K$\alpha_{2}$ Shift [eV]
Cu^+1^	[Ar]3d^10^	7749.3	298.1	7730.2	297.1
Zr^+4^	[Kr]	15,366.0	407.1	15,284.2	404.4
Pd^0^	[Kr]4d^10^	20,709.6	466.2	20,556.5	462.1
Ag^+1^	[Kr]4d^10^	21,686.0	475.5	21,517.5	471.2
Sn^+2^	[Kr]5s^2^4d^10^	24,766.2	503.8	24,543.9	498.5

## Data Availability

The data presented in this study are available on request from the corresponding author.
